# Health literacy levels and its determinants among people with asthma in Malaysian primary healthcare settings: a cross-sectional study

**DOI:** 10.1186/s12889-021-11194-w

**Published:** 2021-06-22

**Authors:** H. Salim, S. Shariff Ghazali, P. Y. Lee, A. T. Cheong, N. H. Harrun, S. Mohamed Isa, H. Pinnock

**Affiliations:** 1 Department of Family Medicine, Faculty of Medicine & Health Sciences, Universiti Putra Malaysia, 43400 Serdang, Selangor Malaysia; 2 NIHR Global Health Research Unit on Respiratory Health (RESPIRE), Usher Institute, The University of Edinburgh, Edinburgh, EH89AG United Kingdom; 3 Malaysian Research Institute on Ageing, Universiti Putra Malaysia, Serdang, 43400 Selangor Malaysia; 4 UM eHealth Unit, Faculty of Medicine, University of Malaya, Jalan Universiti, 50603 Petaling Jaya, Kuala Lumpur Malaysia; 5 Pandamaran Health Clinic, Ministry of Health, Persiaran Raja Muda Musa, Klang, 42000 Selangor Malaysia; 6 Botanik Health Clinic, Ministry of Health, Jalan Langat, Klang, 41200 Selangor Malaysia

**Keywords:** Limited health literacy, Asthma, Low-and-middle-income country, Prevalence

## Abstract

**Background:**

Limited health literacy among people with asthma is associated with poor adherence to self-management activities, thus poor clinical outcomes. This study aimed to determine the prevalence of health literacy level and its determinants among people with asthma in the Malaysian primary healthcare settings.

**Method:**

A cross-sectional study was conducted among participants aged > 18 years with asthma who attended five primary health clinics in Malaysia. Systematic random sampling was employed with a final sample of 550 participants. The questionnaires included the validated Malay version of Health Literacy Scale (HLS) and asthma control questionnaire (ACQ). Statistical analysis was done using SPSS version 25. Multiple logistic regression was performed to determine the determinants for limited health literacy.

**Results:**

The participants mean age of the participants was 48 (SD15.4) years. Most of the participants were women (64%) and of Malay ethnicity (51.1%). Nearly half had a secondary level of education, *n* = 112, (45.8%). Mean duration of asthma diagnosis is 20.6 (SD 15.9) years. More than half (62.5%) had a family history of asthma. About half (50.9%) had uncontrolled asthma, with 87.3% self-rated themselves as having controlled asthma. About a third (29.1%) received education on of asthma action plan, but only 7.1% of these owned a written version an asthma action plan. Limited health literacy accounts for 60.5% of the participants. The significant determinants for limited health literacy included lower educational attainment (*p* < 0.001), family history of asthma (*p* = 0.034), < 20 years duration of asthma diagnosis (*p* = 0.031) and not receiving asthma action plan education (*p* < 0.001).

**Conclusion:**

In this study population, more than half of the people living with asthma were found to have limited health literacy, which was associated with not having received self-management education supported by an asthma action plan. Future interventions should include strategies that ensure they meet the needs of people with limited health literacy.

**Supplementary Information:**

The online version contains supplementary material available at 10.1186/s12889-021-11194-w.

## Background

The burden of long-term conditions (LTCs) is increasing worldwide, and health services in many settings are struggling to provide the best care options for people with these conditions, including asthma. Worldwide, asthma affects about 360 million people, which is expected to increase to 400 million by 2025 [1, 2]. The average prevalence of adults with asthma is 4.5% [3], which continues to increase in the less developed nations, possibly due to a lack of resources within the health system [4, 5]. Asthma is the second leading cause of Disability-Adjusted Life Years (DALYs) (0.9% of total all-cause DALYs) and deaths (0.9% of total all-cause deaths) [6], particularly in low–middle-income countries (LMICs) [7]. In Malaysia, the prevalence of adult asthma was at 4.2%, with 1.2% of deaths related to asthma in 2006 [8]. Among adults with asthma, each year, 20% visited the emergency units for acute exacerbations, with 10% of these being admitted and 27.3% reporting losing six or more workdays [8].

Malaysia has a dual health system - public and private. An additional file summarises Malaysia, its health system and primary care setting (see Additional file 1). Private health services are only available to those who can afford high service fees and/or with private health insurance cover [9]. In the public health sector, services are free with a MYR1 (USD 0.24) co-payment for outpatient services [10]. This charge covers the consultation, investigations, and medicines. While the poor are not exempt from co-payment, the fee-waiver system ensures everyone can access the public health service [11]. Government employees and pensioners, school children and those aged 60 years and above receive these general services in the public primary care clinics for free [12]. Although one published report estimated that a typical primary care clinic average waiting time was 60 min and close to half had a consultation of 11–20 min’ duration [13], the high per capita densities of clinics and workforce in urban areas (2.2 clinics and 15.1 providers per 10, 000 population [9]), longer waiting time and shorter consultation time may be the case in many practices. This is reflected in the concern that demand for subsidised healthcare far outstrips supply in Malaysia and many other LMICs [10, 11].

A population survey in Malaysia indicated that about 35% of the general population have limited health literacy levels [14]. In the asthma context, limited health literacy is associated with improper use of inhalers, poor asthma knowledge [15], and increased utilisation of emergency care and hospitalisation for asthma exacerbations [16, 17]. People with asthma need to interpret their symptoms and act on them, including adhering to medication, adjusting treatment or deciding to seek advice in the event of deterioration [18, 19]. Without the proper support, it may be challenging for people with limited health literacy [20, 21]. A review of health literacy definitions by Sørensen et al. (2012) describes health literacy as an individual’s knowledge, motivation and competence to assess, understand, appraise and apply health information to make healthcare decisions, and exercise disease prevention and health promotion throughout the life course [22]. Factors that mediate limited health literacy among people with asthma include socio-demographic factors, such as income, educational attainment, social support, and employment [16, 23]. Other factors include ethnicity, cultural background and language [23]. These reports may have suggested that socially disadvantaged populations tend to be disproportionately burdened by limited health literacy and the effect on their health. However, more recent literature has shed light on limited health literacy as a marker of broader life circumstances, including but not restricted to limited access to education, limited language proficiency and learning differences [24, 25]. Tailored asthma interventions for people with limited health literacy may improve health outcomes [21] through a co-creation approach [26], embedding technology and creative method [27, 28].

A few studies have looked at the impact of health literacy in Malaysia [29], but none has measured it in people with asthma. Asthma is given less priority than other LTCs in Malaysia such as diabetes, with a lack of funding and attention from a health policy point of view [14]. It has been shown that people with limited health literacy are at risk of not receiving adequate and effective patient care [24]. Understanding the burden of limited health literacy in asthma and highlighting the associated factors can potentially initiate actions to narrow health inequality gaps in asthma care and within the broader public health system [30, 31]. Using a locally validated tool available in the national language [32], we conducted this study to measure the health literacy level among people with asthma in Malaysia and identify its determinants in the Malaysian primary healthcare setting. Our secondary aims include determining the prevalence of smartphones and social media use among people with asthma to identify the potential use of technology in future asthma self-management intervention. Identifying the target group and factors affecting them may facilitate targeted intervention.

## Methods

Ethical approval was obtained from the Medical Research & Ethics Committee, Ministry of Health Malaysia [NMRR-17-1508-36,071].

### Setting

We conducted a cross-sectional study in five selected primary health care clinics in a district in Selangor state, Malaysia, from September 2017 to February 2018. The clinics were purposively chosen to represent urban and suburban settings. All five clinics were headed by at least one trained family physician with 11–26 medical officers, depending on the clinics’ size and the number of patients attending per day. Each doctor could expect to see 50–70 patients daily.

### Study population and recruitment

The study population were adults aged ≥18 years with a physician diagnosis of asthma who attended the outpatient, emergency, and follow-up clinics for any service. Diagnosis of asthma is based on a combination of a history of symptoms typical of asthma (i.e., wheezing, coughing, shortness of breath) and the presence of obstructive airflow reversibility using peak flow variability or spirometry (where available) variability [33]. We excluded those who visited the clinic for acute exacerbations or needing admission and people with cognitive impairments prohibiting informed consent and participation in study data collection. Systematic random sampling was employed. By drawing a lot, the study recruited the first participant with asthma on day one of the data collection, and the participant became the reference point of recruitment. After that, with an interval of 1 out of every two participants, they were approached for recruitment at the registration counters.

### Sample size calculation

The sample size was calculated using the Daniel (2013) [13] formula based on a study by Mancuso et al. (2006) of an 18% limited (marginal/inadequate) health literacy level among the population with asthma [16]. The estimated sample size was 550 after considering a confidence level of 99, 5% precision and 30% non-responding and missing data.

### Study instrument

The study instrument was a pre-tested structured questionnaire. It comprised four elements: socio-demographic, medical information, asthma control questionnaire (ACQ) [34, 35] and the Malay version of Health Literacy Scale (HLS) [32].

Part one consisted of socio-demographic information, including age, sex, ethnic group, marital status, educational attainment, household income, social media use, smartphones, and mobile data plan ownership. Part two consisted of medication information, including duration of asthma diagnosis, use of preventer, any family history of asthma, self-rated asthma control, education on the asthma action plan and own a copy of the written version, other medical problems and body mass index (BMI). The definition of BMI was based on the World Health Organization (WHO) recommendation for the Asian population [36]. Underweight is defined if the BMI < 18.5 kg/m^2^, normal weight is defined if the BMI is 18.5–22.9 kg/m^2^, overweight is defined with a BMI of 23–27.4 kg/m^2^ and obese is defined if the BMI is > 27.5 kg/m^2^.

The asthma control questionnaire (ACQ) is a validated tool to measure asthma treatment’s adequacy [34, 35]. The ACQ has strong measurement properties and has been fully validated for clinical practice and clinical trials. The ACQ has strong discriminative and evaluative properties; it can detect small differences between patients with different asthma control levels, and it is very sensitive to within-patient change in asthma control over time [34, 35, 37]. Patients with a score below 1.0 will have adequately controlled asthma, and above 1.0, their asthma will not be well controlled. However, to be confident that a patient has well-controlled asthma, the optimal cut-point is 0.75 (negative predictive value = 0.85) and with inadequately controlled asthma, the optimal cut-point is 1.50 (positive predictive value = 0.88) [38]. The short version ACQ (symptoms alone-ACQ version) was used. This questionnaire assesses the patients’ asthma control by asking for patients’ experiences during the previous week in response to the five questions (night-time waking, symptoms on waking, activity limitation, shortness of breath, and wheezing). A 7-point scale is used to grade the severity level (0 = no impairment; 6 = maximum impairment) [35]. There is a good internal consistency of these five questions (Cronbach *α* = 0.98). It measures the same construct as the original ACQ, and the agreement between this short version ACQ and the original ACQ is high (Interclass correlation, ICC = 0.94) [35].

The HLS contains 47 items measuring health literacy [32, 38]. The HLS is based on a conceptual model of health literacy and measures four competencies to deal with health-relevant information (access/obtain, understand, appraise/judge/evaluate, and apply/use health information) in three domains with a good internal consistency of 0.97 [38]. The three domains are health care, disease prevention, and health promotion [38]. Each item’s perceived difficulty is rated on a 4-point Likert scale (1 = very difficult, 2 = difficult, 3 = easy, and 4 = very easy), with a possible lowest mean score of 1 and a possible highest mean score of 4. The raw scores of the 47 items of the HLS-Q47 are used to generate an ‘Index’ with defined levels for dichotomised categories of ‘limited’ or ‘adequate’ health literacy levels [38]. Limited health literacy is defined as an *Index* < 33 points. This threshold was set according to an expert assessment of the health literacy scores that increased the likelihood of a person successfully pursuing their own health interest [38]. The tool was translated into Malay language and is adjudged reliable with Cronbach *α* of 0.96 [32]. Experienced public health researchers in Malaysia pre-tested the questionnaire for readability and understandability, and the content was verified to reflect cultural perspectives [32].

### Data collection

Those interested in taking part were informed about the study, and written consent was obtained before participating in this study. The demographic questionnaire was self-administered, and medical data were verified from the medical record. The HLS and ACQ were interviewer-assisted. Research assistants assisted in completing the entire questionnaires for anyone who needed it. Two research assistants were stationed at each clinic, where training to use the tools and verifying medical information from the patient’s asthma book were conducted before the study commenced, and a refresher training delivered in the middle of the data collection period for each clinic by the principal investigator. The research assistants were medical graduates awaiting their internships. In this district, every asthma patient has an asthma book, a duplicate of the practice’s medical records. All questionnaires were available in the Malay language. All healthcare staff were informed regarding the conduct of the study.

### Data analysis

Statistical analysis was done using SPSS version 25.0. Descriptive statistics were used to describe the participants’ demographic and disease characteristics and their health literacy level. Percentages and frequencies were used for the categorical variables, while mean and standard deviations were calculated for the continuous variables. The associations for numerical data were tested using chi-square tests. Simple logistic regressions were performed, and factors with a *p*-value < 0.2 were included in the multiple logistic regression model. Multiple logistic regression was performed to identify the determinants for limited health literacy. The model results were presented as beta coefficient, standard error, odds ratio and 95% confidence intervals. A significant level was set at a p-value < 0.05.

## Results

**Fig. 1:**
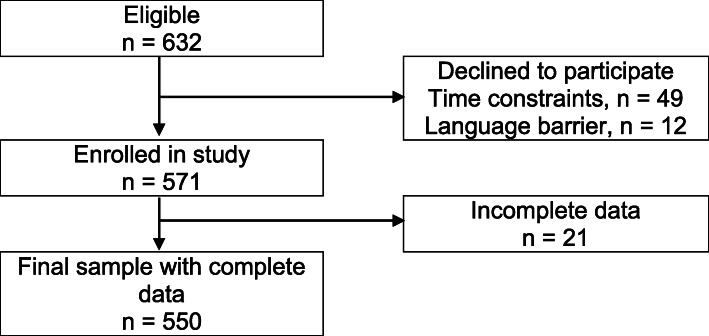
Flowchart of participant recruitment

The study finally included 550 participants giving a response rate of 87% (550/632). We summarised the recruitment process using the flowchart in Fig. 1.

### Basic characteristics

Table 1 shows the characteristics of the study participants. The mean age of the study participants was 48 (SD15.44) years. More than two-thirds (69.3%) of study participants were in the age range of 40 years and above, and just a third were males (36%). Approximately half (51.1%) were from the Malay ethnic group. The majority (71.1%) were not married. Two-thirds of the study population had secondary education and above (66.7%). However, the majority were in the low household income group (83.3%).

**Table 1:** Socio-demographic, disease characteristics, and associations

Variables	All patients *N* = 550	Adequate health literacy *N* = 221		Limited health literacy *n* = 329		Significance
		N	%	n	%	
**Age**	Mean (SD): 48 (15.44)					< 0.001*
< 40	30.7%	87	39.4	82	24.9	
> 40	69.3%	134	60.6	247	75.1	
**Sex**						0.777
Male	36.0%	78	35.3	120	36.5	
Female	64.0%	143	64.7	209	63.5	
**Ethnicity**						0.208
Chinese	12.4%	24	10.9	44	13.4	
Malay	51.1%	123	55.7	158	48.0	
Indian	36.5%	74	33.5	127	38.6	
**Marital status**						0.058
Not married	28.9%	59	26.7	65	19.8	
Married	71.1%	162	73.3	264	80.2	
**Educational attainment**						< 0.001*
No formal education/primary education	33.3%	34	15	149	45.3	
Secondary education	45.8%	112	51	140	42.6	
Tertiary education	20.9%	75	34	40	12.2	
**Household income**						< 0.001*
< 3000	83.3%	167	75.6	291	88.4	
> 3000	16.7%	54	24.4	38	11.6	
**Duration of asthma diagnosis (years)**	Mean (SD) 20.6 (15.9)					0.005*
< 20	50.5%	128	57.9	150	45.6	
> 20	49.5%	93	42.1	179	54.4	
**Use of preventer**						0.020*
No	30.2%	79	35.7	87	26.4	
Yes	69.8%	142	64.3	242	73.6	
**Family history of asthma**						0.004*
No	37.5%	67	30.3	139	42.2	
Yes	62.5%	154	69.7	190	57.8	
**Asthma control (based on ACQ)**						0.098
Uncontrolled	50.9%	103	46.6	177	53.8	
Controlled	49.1%	118	53.4	152	46.2	
**Self-rated asthma control**						0.455
Uncontrolled	12.7%	31	14.0	39	11.9	
Controlled	87.3%	190	86.0	290	88.1	
**Education on the asthma action plan**						0.001*
No	70.9%	174	78.7	216	65.7	
Yes (*n* = 160)	29.1%	47	21.3	113	34.3	
-Verbal	22%	19	3.5	102	18.5	
-Written	7.1%	28	5.1	11	2	
**Other medical problems**						0.027*
No	54.9%	134	60.6	168	51.1	
Yes	45.1%	87	39.4	161	48.9	
**Body mass index (kg/m**^**2**^**)**	Mean (SD) = 26.8 (6.4)					0.071
Underweight (< 18.5)	4.7	11	5.0	15	4.6	
Normal weight (18.5–22.9)	26	49	22.2	94	28.6	
Overweight (23–27.4)	30	60	27.1	105	31.9	
Obese (> 27.5)	39.3	101	45.7	115	35.0	

Univariate analysis (Chi-square test). * *P*-value < 0.05

As for disease characteristics, about half (50.5%) had been diagnosed with asthma for less than 20 years. About a third (37.5%) had no family history of asthma. Half (50.9%) of the study participants had uncontrolled asthma with a high rate of preventer use (69.8%); however, the majority (87.3%) self-rated their asthma control as being controlled. About a third have received education on an asthma action plan (29.1%). Of those who answered yes, only 7% of the total population received it in written format (written asthma action plan). About half (54.9%) had no other medical problem. More than half (69.3%) had a BMI of more than 23 kg/m^2^.

Regarding the use of technology and social media (Table 2), slightly more than three-quarters of the participants owned smartphones (78.4%), and almost half owned an internet data plan (48.4%). Half of the study population used social media (50.9%). The leading social media platforms used included Facebook and WhatsApp while other social media platforms included Instagram, Twitter, Telegram, and WeChat. Facebook and WhatsApp had equal numbers of users from both age groups, 44.1% in the under 40 years cohort and 55.9% in the 40 years and over cohort. The majority of Instagram and Twitter users were in the under 40 age group, 76 and 80%, respectively.

**Table 2:** Digital technology and use of social media

Variables	Results, N (%)	
**Smartphone ownership**		
No	119	21.6
Yes	431	78.4
**Internet data plan ownership**		
No	284	51.6
Yes	266	48.4
**Use of social media**		
No	270	49.1
Yes	280	50.9

### Levels of health literacy and associated factors

More than half (60.5%, *n* = 333) had limited health literacy in assessing health literacy at two levels. Table 1 shows the associations between limited health literacy and the socio-demographic and disease characteristics of study participants. Socio-demographic factors that were associated with limited health literacy included age (*p* < 0.001), educational attainment (p < 0.001) and household income (p < 0.001). For disease characteristics, factors that were associated with limited health literacy included duration of asthma diagnosis (*p* = 0.005), use of preventer (*p* = 0.020), any family history of asthma (*p* = 0.004), ownership of action plan (*p* = 0.001) and other medical problems (*p* = 0.027).

All variables with a *p*-value of 0.200 and less were included in the multiple logistic regression modelling (Table 3). As demonstrated in Table 3, educational attainment, a family history of asthma, duration of asthma diagnosis and ownership of asthma action plans were predictors of limited health literacy.

**Table 3:** Multiple logistic regression model for the determinants of health literacy categories

Variables	Beta	SE	Odds ratio	95% CI		P-value
				Lower	Upper	
**Age**						
< 40	Ref					
> 40	−0.119	0.242	0.888	0.553	1.425	0.621
**Educational attainment**						
No formal education or primary education	Ref	–	–	–	–	–
Secondary level	−1.200	0.234	0.301	0.190	0.476	< 0.001^*^
Tertiary level	−2.184	0.287	0.113	0.064	0.198	< 0.001^*^
**Household income**						
< 3000	Ref	–	–	–	–	–
> 3000	0.275	0.274	1.317	0.769	2.254	0.316
**On preventer medications**						
No	Ref	–	–	–	–	–
Yes	0.001	0.215	1.001	0.657	1.525	0.996
**Family history of asthma**						
No	Ref	–	–	–	–	–
Yes	0.426	0.202	1.532	1.032	2.274	0.034^*^
**Other medical condition**						
No	Ref	–	–	–	–	–
Yes	−0.113	0.212	0.893	0.590	1.353	0.594
**Duration of asthma (years)**						
< 20	Ref	–	–	–	–	–
> 20	−0.416	0.193	0.659	0.452	0.963	0.031^*^
**Education on the asthma action plan**						
No	Ref	–	–	–	–	–
Yes	−0.912	0.225	0.402	0.259	0.624	< 0.001^*^

Notes: *SE* standard errors, *CI* confidence interval; *p*-value significant of less than 0.05^*^, *Ref* reference group

Attaining a secondary (B − 1.20, OR 0.3, *P*-value < 0.001, 95% CI 0.19 to 0.48,) and tertiary (B − 2.18, OR 0.1, *P*-value < 0.001, 95% CI 0.06 to 0.20) level of education reduces the probability of a person with asthma having limited health literacy by 30.1 and 11.3%, respectively. Those with a family history of asthma (B 0.43, OR 1.5, *P*-value 0.03, 95% CI 1.03 to 2.27) were 1.5 times more likely to have limited health literacy. For having asthma for 20 years and more (B − 0.42, OR 0.66, P-value 0.03, 95% CI 0.45 to 0.96), there was a 66% reduction in the probability of a person with asthma having limited health literacy. Those who received education on an asthma action plan (B − 0.91, OR 0.4, P-value < 0.001, 95% CI 0.26 to 0.62), is associated with a reduced probability of having limited health literacy by 40.2%.

## Discussion

### Summary of findings

In this study, we aimed to determine the prevalence of limited health literacy and the factors influencing the health literacy level among people with asthma in the primary health clinics in Malaysia’s central state. Overall, we found that 60.5% of people with asthma have limited health literacy, particularly in disease prevention and health promotion. Half of the study participants had uncontrolled asthma despite a high rate of preventer use. The limited health literacy level was associated with i) educational attainment; no formal education or only primary education were associated with limited health literacy, ii) family history of asthma; a positive family history was associated with limited health literacy, iii) the duration of asthma diagnosis; duration of asthma of less than 20 years is associated with limited health literacy and iv) education on an asthma action plan; not receiving education on an asthma action plan is associated with limited health literacy.

### Interpretation of the findings and comparison with previous findings

In this study, nearly two-thirds of people with asthma (60.5%) had limited health literacy. The prevalence of limited health literacy in this study was compared to an estimate of 35% in a population study on health literacy [14]. While we used the HLS-Q47 among asthma patients in the primary care settings, the population study used the HLS-Q18 among the general Malaysian population [14]. Globally, only a few studies examine the prevalence of limited health literacy and its associated factors among people with asthma. Many of these studies are concentrated in high-income countries such as the United States (7–35%) [15, 16, 39] and Australia (45%) [17]. However, in all of these studies, the use of different assessment tools and population sampling technique may make it difficult to compare the results, though it is apparent that limited health literacy is a public health challenge in many health settings.

This study found that educational attainment is associated with limited health literacy, echoing findings elsewhere in the literature [16, 23]. Although limited health literacy is associated with a range of socio-demographic characteristics, it tends to affect vulnerable populations disproportionately, including people with lower educational attainment, of lower socioeconomic status, people from ethnic minorities, and those whose spoken language differs from the majority population. It has been argued that health literacy may not fully explain the broader relationships connecting it to social determinants of health and health outcomes [24]. Health literacy should not be regarded as a single characteristic but rather as a marker for multiple life circumstances, such as systemic and socio-cultural challenges, that contribute to limited health literacy [24]. For example, health information in Malaysia is printed in Malay and English languages. However, many people in this multilingual society speak other languages, such as Mandarin and Tamil, which may hinder their access to the health information on disease promotion and prevention. Despite being a multilingual nation, limited interpreter services have continued to be a problem, echoing challenges in other health settings [40, 41]. Social vulnerability is influenced by how society and the system are constructed, rather than being an inherent characteristic of individual or sub-populations [24], and culturally tailored interventions exert a considerable influence on health literacy [28, 42]. Nonetheless, defining health literacy at the disease level, as in the case of asthma, is a step toward raising awareness of the issue and dismantling practices, structures, and policies that may have contributed to exacerbating health disparities in asthma care in Malaysia and other LMICs [30].

In this study, we found that having a positive family history for asthma was linked to limited health literacy, which may appear to contradict literature in other disease contexts [43, 44]. It has previously been reported that awareness within a family of a chronic disease may act as an impetus to gain more knowledge about the condition and how to cope with it [43]. People living with a chronic condition often drew on the health literacy skills of others in their family to understand and use health information [45], a practice which might be expected to be amplified in the context of a condition (such as asthma) with a strong family history. However, our contradictory findings in this study suggest that the inter-relationship of family history and health literacy is multifactorial and influenced, for example, by social-economic context. Further research will be needed to understand how family history of asthma shapes health literacy.

### The role of health literacy on asthma control and self-management

Although other studies did not find an association between duration of asthma diagnosis and health literacy level [16, 17], we found that a longer duration of asthma diagnosis reduced the probability of a person having limited health literacy. It may have been that the patients who have more extended engagement with the health system were more aware of general health information, including health promotion and disease prevention. The engagement can be in the form of scheduled and unscheduled visits for asthma. Studies have shown that people with limited health literacy had a higher emergency use rate and hospitalisations due to severe exacerbations [15–17]. In this study, almost half of the participants with limited health literacy had uncontrolled asthma despite a high preventer use rate. However, more than two-thirds of people with limited health literacy self-rated their asthma as controlled. A mismatch of a control definition is common in the literature between healthcare professionals and people with asthma [46]. The mismatch in the understanding of asthma control may challenge people’s ability to self-manage their asthma, especially people with limited health literacy.

We found that those who received education on the asthma action plan is associated with a lesser probability of having limited health literacy. Overall, a higher number of participants with limited health literacy in this study received education on an asthma action plan compared to those with adequate health literacy. This situation may have come about because more of them had uncontrolled asthma, thus, education on the action during an exacerbation was more likely to be emphasised during unscheduled visits. In this study, only a small number of people received a written asthma action plan and even fewer people with limited health literacy. Supported asthma self-management in written asthma action plans and regular review improves health outcomes and reduces mortality [47, 48]. It may have been that practitioners recognised patients’ challenges with understanding health information and assumed that patients have limited health literacy, thus, they may not benefit from a written action plan if prescribed one. Practitioners may have underestimated the role of tailored support for patients’ needs [49] as people with limited health literacy have as much right to supported asthma self-management as any other patients.

Health literacy skills equipped a person with the knowledge of how to act and the ability to decide when to seek treatment during an exacerbation. Enabling asthma self-management by creating awareness about asthma action plans and adopting one is a challenge in many settings. Due to the high rate of smartphone ownership, and much use of data plans and social media among the participants in this study, digital technologies may be utilised in future asthma self-management intervention for people with limited health literacy [9].

### Strengths and limitations

This study was conducted in government primary care clinics, the leading provider of chronic disease care. We collected the data from multiple sites covering centers from the urban and suburban areas. However, this study does have a few limitations. As this is a cross-sectional study, causality could not be determined. Nevertheless, the associations found in this study may provide a ground for future targeted interventions employing health literacy to improve asthma care and health inequality in the region. In the context of low socioeconomic status among the participants in this study, social desirability and response bias may have underestimated the true limited level of health literacy. The HLS used in this study only assessed general health literacy and may not have accurately reflected participants’ specific understanding of their asthma. Using health literacy as a binary variable remains a flaw, especially since health literacy is a spectrum that interacts in complex ways with the wider environment and socio-cultural factors. Health literacy is not linearly related to health outcomes but influences other healthcare aspects, including self-management [50] and may be mediated by other circumstances or challenges in the system, such as access to education [24]. Limited health literacy should not be viewed as an individual’s problem but as a public health issue and a barrier to providing adequate care for all. Despite the importance of disease severity, quality of life and medication adherence information, we limited the questionnaires used to HLS-Q47 and ACQ to answer specific research questions whilst improving participation and maximising final analysis response. Data collection was not formally audited, but all data collectors were trained to use the tools and verify medical information from the patient’s asthma records, and a further training exercise took place at each clinic.

### Implications for practice, research, and policy

Health literacy is a public health burden in many health care systems, and limited health literacy may affect the adoption of guideline-recommended self-management practices. Limited health literacy may be influenced by a variety of factors, and a quantitative assessment of health literacy may not provide a complete picture of the problem. To identify specific areas to target for future interventions, tools that measure reading ability and other functional skills such as comprehension and numeracy may be required first. Further research is needed to gain a comprehensive understanding of the situation and to develop appropriately targeted interventions that address the nuanced socio-demographic context of a multicultural and multilingual society. However, it is a step forward to highlight the problem and create awareness within the health system itself, i.e., healthcare professionals. Future efforts to understand the practitioner’s perspective about the tailored prescription of written action plans in this setting is crucial to improving its delivery. There is a need for researchers, particularly in public health, to explore and understand the role of socio-cultural ecology, i.e., family relationships and societal practices and the attendant influence on improving health literacy among people with asthma. Policy-makers should focus on cost-effective efforts to reduce socioeconomic deprivation and health inequality among people with limited health literacy and asthma.

## Conclusion

This study described the dearth of literature on health literacy among people with asthma in a multi-ethnic Malaysia. Overall, the limited health literacy level among people with asthma is high. Lower educational attainment, a positive family history, a shorter duration of asthma diagnosis and not receiving education on an asthma action plan are associated with limited health literacy. We highlighted the gap in asthma and self-management status in an LMIC setting. Identification of specific areas (i.e., functional skills, language and social practices) to target for future intervention is needed. The results of this study will help to inform the public health authority locally and will be of interest globally.

## Supplementary Information

**Additional file 1.**

## Data Availability

The datasets used and/or analysed during the current study are available from the corresponding author on reasonable request. The dataset that supports the conclusions is available within the manuscript.

## Abbreviations

HLS: Health Literacy Scale
ACQ: asthma control questionnaire
LTCs: long-term conditions
LMICs: low- and middle-income countries
ICC: interclass correlation
BMI: body mass index
WHO: World Health Organization

## References

[1] Masoli M, Fabian D, Holt S, Beasley R (2004). Global initiative for asthma program. The global burden of asthma: executive summary of the GINA dissemination committee report. Allergy..

[2] GBD 2015 Chronic Respiratory Disease Collaborators (2017). Global, regional, and national deaths, prevalence, disability-adjusted life years, and years lived with disability for chronic obstructive pulmonary disease and asthma, 1990–2015: a systematic analysis for the Global Burden of Disease Study 2015. Lancet Respir Med.

[3] Stanojevic S, Moores G, Gershon AS, Bateman ED, Cruz AA, To T (2012). Global asthma prevalence in adults: findings from the cross-sectional world health survey. BMC Public Health.

[4] Lundbäck B, Backman H, Lötvall J, Rönmark E (2016). Is asthma prevalence still increasing?. Expert Rev Respir Med.

[5] Cruz ÁA, Stelmach R, Ponte EV (2017). Asthma prevalence and severity in low-resource communities. Curr Opin Allergy Clin Immunol.

[6] Pinnock H, Parke HL, Panagioti M, Daines L, Pearce G, Epiphaniou E (2017). Systematic meta-review of supported self-management for asthma: a healthcare perspective. BMC Med.

[7] Beran D, Zar HJ, Perrin C, Menezes AM, Burney P (2015). Forum of international respiratory societies working group collaboration. Burden of asthma and chronic obstructive pulmonary disease and access to essential medicines in low-income and middle-income countries. Lancet Respir Med.

[8] National Health and Morbidity Survey 2006. http://www.iku.gov.my/images/IKU/Document/REPORT/2006/Asthma.pdf. Accessed 25 September 2020.

[9] Lim HM, Sivasampu S, Khoo EM, Mohamad NK (2017). Chasm in primary care provision in a universal health system: findings from a nationally representative survey of health facilities in Malaysia. PLoS One.

[10] Tangcharoensathien V, Patcharanarumol W, Ir P, Aljunid SM, Mukti AG, Akkhavong K, Banzon E, Huong DB, Thabrany H, Mills A (2011). Health-financing reforms in Southeast Asia: challenges in achieving universal coverage. Lancet..

[11] van Doorslaer E, O'Donnell O, Rannan-Eliya RP, Somanathan A, Adhikari SR, Garg CC, Harbianto D, Herrin AN, Huq MN, Ibragimova S, Karan A, Lee TJ, Leung GM, Lu JFR, Ng CW, Pande BR, Racelis R, Tao S, Tin K, Tisayaticom K, Trisnantoro L, Vasavid C, Zhao Y (2007). Catastrophic payments for health care in Asia. Health Econ.

[12] Fadzil F, Jaafar S, Ismail R (2020). 40 years of Alma Ata Malaysia: targeting equitable access through organisational and physical adaptations in the delivery of public sector primary care. Prim Health Care Res Dev.

[13] Ahmad BA, Khairatul K, Farnaza A (2017). An assessment of patient waiting and consultation time in a primary healthcare clinic. Malays Fam Physician.

[14] National Health and Morbidity Survey 2019. http://www.iku.gov.my/images/IKU/Document/REPORT/NHMS2019/Report_NHMS2019-NCD_v2.pdf. Accessed 25 September 2020.

[15] Apter AJ, Wan F, Reisine S, Bender B, Rand C, Bogen DK, Bennett IM, Bryant-Stephens T, Roy J, Gonzalez R, Priolo C, Have TT, Morales KH (2013). The association of health literacy with adherence and outcomes in moderate-severe asthma. J Allergy Clin Immunol.

[16] Mancuso CA, Rincon M (2006). Impact of health literacy on longitudinal asthma outcomes. J Gen Intern Med.

[17] Adams RJ, Appleton SL, Hill CL, Ruffin RE, Wilson DH (2009). Inadequate health literacy is associated with increased asthma morbidity in a population sample. J Allergy Clin Immunol.

[18] Londoño AM, Schulz PJ (2014). Impact of patients' judgment skills on asthma self-management: a pilot study. J Public Health Res.

[19] Apter AJ, Wang X, Bogen D, Bennett IM, Jennings RM, Garcia L, Sharpe T, Frazier C, ten Have T (2009). Linking numeracy and asthma-related quality of life. Patient Educ Couns.

[20] Federman AD, Wolf MS, Sofianou A, O'Conor R, Martynenko M, Halm EA (2014). Asthma outcomes are poor among older adults with low health literacy. J Asthma.

[21] Sheridan SL, Halpern DJ, Viera AJ, Berkman ND, Donahue KE, Crotty K (2011). Interventions for individuals with low health literacy: a systematic review. J Health Commun.

[22] Sørensen K, Van den Broucke S, Fullam J, Doyle G, Pelikan J, Slonska Z (2012). Health literacy and public health: a systematic review and integration of definitions and models. BMC Public Health.

[23] Seibert RG, Winter MR, Cabral HJ, Wolf MS, Curtis LM, Paasche-Orlow MK (2019). Health literacy and income mediate racial/ethnic asthma disparities. Health Literacy Res Pract.

[24] Schillinger D. The intersections Between social determinants of health, health literacy, and health disparities. Stud Health Technol Inform. 2020:269, 22–41. 10.3233/SHTI200020 PMID: 32593981; PMCID: PMC7710382. 10.3233/SHTI200020 PMC7710382 32593981

[25] Stormacq C, Van den Broucke S, Wosinski J (2019). Does health literacy mediate the relationship between socioeconomic status and health disparities? Integrative review. Health Promot Int.

[26] Nash S, Arora A (2021). Interventions to improve health literacy among Aboriginal and Torres Strait islander peoples: a systematic review. BMC Public Health.

[27] Meherali S, Punjani NS, Mevawala A (2020). Health literacy interventions to improve health outcomes in low- and middle-income countries. Health Literacy Res Pract..

[28] Sobel RM, Paasche-Orlow MK, Waite KR, Rittner SS, Wilson EA, Wolf MS (2009). Asthma 1-2-3: a low literacy multimedia tool to educate African American adults about asthma. J Commun Health.

[29] Abdullah A, Liew SM, Salim HS, Ng CJ, Chinna K (2020). Health literacy research in Malaysia: a scoping review. Sains Malays.

[30] Canino G, McQuaid EL, Rand CS (2009). Addressing asthma health disparities: a multilevel challenge. J Allergy Clin Immunol.

[31] Dunn P, Conard S (2018). Improving health literacy in patients with chronic conditions: a call to action. Int J Cardiol.

[32] Duong TV, Aringazina A, Baisunova G, Nurjanah PTV, Pham TV, Pham KM (2017). Measuring health literacy in Asia: validation of the HLS-EU-Q47 survey tool in six Asian countries. J Epidemiol.

[33] Ban A, Omar A, Chong LY, Lockman H, Ida Zaliza ZA, Ali I (2018). Management of asthma in adults in primary care. Malays Fam Physician.

[34] Juniper E, O’byrne P, Guyatt G, Ferrie P, King D (1999). Development and validation of a questionnaire to measure asthma control. Eur Respir.

[35] Juniper EF, Svensson K, Mörk AC, Ståhl E (2005). Measurement properties and interpretation of three shortened versions of the asthma control questionnaire. Respir Med.

[36] WHO Expert Consultation (2004). Appropriate body-mass index for Asian populations and its implications for policy and intervention strategies. Lancet..

[37] Juniper EF, Bousquet J, Abetz L, Bateman ED, GOAL Committee (2006). Identifying ‘well-controlled’ and ‘not well-controlled’ asthma using the Asthma Control Questionnaire. Respir Med.

[38] Sørensen K, Van den Broucke S, Pelikan JM, Fullam J, Doyle G, Slonska Z (2013). Measuring health literacy in populations: illuminating the design and development process of the European health literacy survey questionnaire (HLS-EU-Q). BMC Public Health.

[39] Federman AD, Wisnivesky JP, Wolf MS, Leventhal H, Halm EA (2010). Inadequate health literacy is associated with suboptimal health beliefs in older asthmatics. J Asthma..

[40] Poureslami I, Rootman I, Doyle-Waters MM, Nimmon L, Fitzgerald JM (2011). Health literacy, language, and ethnicity-related factors in newcomer asthma patients to Canada: a qualitative study. J Immigr Minor Health.

[41] Hughson JA, Marshall F, Daly JO, Woodward-Kron R, Hajek J, Story D (2018). Health professionals' views on health literacy issues for culturally and linguistically diverse women in maternity care: barriers, enablers and the need for an integrated approach. Aust Health Rev.

[42] Poureslami I, Nimmon L, Doyle-Waters M, Rootman I, Schulzer M, Kuramoto L, FitzGerald JM (2012). Effectiveness of educational interventions on asthma self-management in Punjabi and Chinese asthma patients: a randomised controlled trial. J Asthma..

[43] McKenna VB, Sixsmith J, Barry MM (2017). The relevance of context in understanding health literacy skills: findings from a qualitative study. Health Expect.

[44] Rowlands G, Shaw A, Jaswal S, Smith S, Harpham T (2017). Health literacy and the social determinants of health: a qualitative model from adult learners. Health Promot Int.

[45] Edwards M, Wood F, Davies M, Edwards A (2015). 'Distributed health literacy': longitudinal qualitative analysis of the roles of health literacy mediators and social networks of people living with a long-term health condition. Health Expect.

[46] Price D, David-Wang A, Cho SH, Ho JC, Jeong JW, Liam CK (2015). Time for a new language for asthma control: results from REALISE Asia. J Asthma Allergy.

[47] Pearce G, Parke HL, Pinnock H, Epiphaniou E, Bourne CL, Sheikh A (2016). The PRISMS taxonomy of self-management support: derivation of a novel taxonomy and initial testing of its utility. J Health Serv Res Policy.

[48] Levy ML (2014). National Review of asthma deaths (NRAD). Br J Gen Pract.

[49] Melton C, Graff C, Holmes GN, Brown L, Bailey J (2014). Health literacy and asthma management among African-American adults: an interpretative phenomenological analysis. J Asthma..

[50] Paasche-Orlow MK, Wolf MS (2007). The causal pathways linking health literacy to health outcomes. Am J Health Behav.

