# Supplementary material for: Health literacy levels and its determinants among people with asthma in Malaysian primary healthcare settings: a cross-sectional study
Source: BMC Public Health. 2021 Jun 22;21:1186. doi: 10.1186/s12889-021-11194-w (PMC8218499; doi:10.1186/s12889-021-11194-w)
Supplement: Supplementary file 1 — Additional file 1. [file 12889_2021_11194_MOESM1_ESM.docx]

Additional file 1 Summary about Malaysia, its health system and the primary care service.

| **Malaysia, the health system and primary care setting.** |
| --- |
| - Malaysia is a multiracial country comprising three main ethnic groups: *Bumiputera* (Malay and Indigenous group) (65.1%), Chinese (26%) and Indian (7.7%) [1]. |
| - The national language, *Bahasa Malaysia*, is used as the main medium of instruction in both primary and secondary national schools. |
| - The Government provides primary, secondary, and tertiary care for the population. |
| - A typical primary health care clinic consists of a family medicine specialist, medical officers, paramedics, nurses, and other allied health practitioners such as a pharmacist, dietitian, and/or nutritionist, physiotherapist, occupational therapist, laboratory and radiology technicians and medical social workers [2]. |
| - The primary care clinics provide comprehensive care, which includes acute and chronic care, prevention and health promotion services, rehabilitation, and palliative care services, maternal and child health, and dental care [2]. |
| - The Government supports the public health sector through taxation with a very low co-payment for those of working age. The private sector is a 'fee-for-service' model often covered by private health insurance policies. |

**References:**

1. Department of Statistics. Population distribution and basic demographic characteristic - Census 2000. Available: [https://www.mycensus.gov.my/index.php/census-product/publication/census-2000/650-population-distribution-and-basic-demographic-characteristics-2000. Accessed 20 September 2020](https://www.mycensus.gov.my/index.php/census-product/publication/census-2000/650-population-distribution-and-basic-demographic-characteristics-2000.%20Accessed%2020%20September%202020).
2. Fadzil F, Jaafar S, Ismail R. 40 years of Alma Ata Malaysia: targeting equitable access through organisational and physical adaptations in the delivery of public sector primary care. Prim Health Care Res Dev. 2020;21:e4. https://doi.org/10.1017/S146342362000002X.
